# Preconditioning of mesenchymal stem cells for improved transplantation efficacy in recessive dystrophic epidermolysis bullosa

**DOI:** 10.1186/scrt511

**Published:** 2014-11-06

**Authors:** Christopher Perdoni, John A McGrath, Jakub Tolar

**Affiliations:** Department of Pediatrics, Stem Cell Institute & Division of Blood and Marrow Transplantation, 420 Delaware St SE, MMC 366, Minneapolis, MN 55455 USA; St. John’s Institute of Dermatology, King’s College, London (Guy’s Campus), Strand, London, England, WC2R 2LS United Kingdom

## Abstract

**Introduction:**

The use of hematopoietic cell transplantation (HCT) has previously been shown to ameliorate cutaneous blistering in pediatric patients with recessive dystrophic epidermolysis bullosa (RDEB), an inherited skin disorder that results from loss-of-function mutations in *COL7A1* and manifests as deficient or absent type VII collagen protein (C7) within the epidermal basement membrane. Mesenchymal stem cells (MSCs) found within the HCT graft are believed to be partially responsible for this amelioration, in part due to their intrinsic immunomodulatory and trophic properties and also because they have been shown to restore C7 protein following intradermal injections in models of RDEB. However, MSCs have not yet been demonstrated to improve disease severity as a stand-alone systemic infusion therapy. Improving the efficacy and functional utility of MSCs via a pre-transplant conditioning regimen may bring systemic MSC infusions closer to clinical practice.

**Methods:**

MSCs were isolated from 2- to 4-week-old mice and treated with varying concentrations of transforming growth factor-β (TGFβ; 5-20 ng/mL), tumor necrosis factor- α (TNFα; 10-40 ng/mL), and stromal cell-derived factor 1-α (SDF-1α; 30 ng/mL) for 24-72 hours.

**Results:**

We demonstrate that treating murine MSCs with exogenous TGFβ (15 ng/mL) and TNFα (30 ng/mL) for 48 hours induces an 8-fold increase in *Col7a1* expression and a significant increase in secretion of C7 protein, and that the effects of these cytokines are both time and concentration dependent. This cytokine treatment also promotes a 4-fold increase in *Tsg-6* expression, a gene whose product is associated with improved wound-healing and immunosuppressive features. Finally, the addition of exogenous SDF-1α to this regimen induces a simultaneous upregulation of *Col7a1, Tsg-6*, and *Cxcr4* expression.

**Conclusions:**

These data suggest that preconditioning represents a feasible method for improving the functional utility of MSCs in the context of RDEB stem cell transplantation, and also highlight the applicability of preconditioning principles toward other cell-based therapies aimed at treating RDEB patients.

## Introduction

Epidermolysis bullosa represents a spectrum of blistering diseases that vary in genetic etiology, molecular phenotype, and clinical severity [1]. Of the major epidermolysis bullosa subtypes, one of the most profound in terms of clinical presentation and progression is recessive dystrophic epidermolysis bullosa (RDEB). RDEB is characterized by loss-of-function mutations within the collagen type VII gene (*COL7A1*), ultimately manifesting as the reduced presence of type VII collagen protein (C7) within the dermal–epidermal junction (DEJ) [2, 3]. Normally, C7 is synthesized and secreted as procollagen homotrimers by keratinocytes and dermal fibroblasts, and further processed and assembled within the extracellular space into anti-parallel dimers, which polymerize into anchoring fibrils [3, 4]. Anchoring fibrils provide a structural attachment between the epidermal basement membrane and papillary dermis, thus strengthening the DEJ [5]. In RDEB, however, the diminished presence of functional C7 precludes DEJ integrity and results in the blisters and erosions seen clinically.

Various strategies have emerged with regards to approaching RDEB therapy, including genetic correction of RDEB cells [6–9], intradermal injection of allogeneic fibroblasts [10, 11], as well as intradermal [12, 13] and systemic [14] injection of recombinant C7. While intradermal fibroblast injections have been shown to improve wound healing in selected areas of ulcerated human RDEB skin, the remaining techniques have yet to be tested in RDEB patients, and none have looked at systemic responses in these populations. In contrast, the use of hematopoietic cell transplantation (HCT) has been demonstrated to promote systemic wound healing and to ameliorate the disease phenotype in pediatric RDEB patients [15, 16]. Specifically, healthy allogeneic donor cells contained within the hematopoietic graft are capable of homing to the site of mucocutaneous injury, engrafting, and promoting repair at wounded recipient tissue sites [17]. However, taking into consideration that RDEB patients are already hypersensitive to infections due to the loss of mucocutaneous barriers, the immunomyeloablative conditioning regimens required for transplantation may exacerbate these predispositions while also introducing additional risks [16].

Reducing the degree of immunomyeloablative conditioning used for transplantation must be weighed against the patient’s likelihood of developing graft-versus-host disease, a major immune complication associated with HCT. A potential solution to this balancing act involves the use of nonhematopoietic mesenchymal stem cells (MSCs), which not only exhibit local immunosuppressive properties [18, 19] but also serve as secretory sources for adhesive molecules, anti-apoptotic and anti-fibrotic growth factors at injured tissue, and other bioactive molecules that support local progenitor cells [20–22]. The co-administration of MSCs within HCT protocols has previously been shown to promote hematopoietic engraftment in the settings of hematologic malignancy [23] and recovery from breast cancer chemotherapy [24], while infusions of MSCs alone have shown additive benefit relative to HCT in the context of osteogenesis imperfecta [25]. Additionally, while the exact mechanisms involved in HCT-mediated amelioration of RDEB are not completely understood, it is thought that nonhematopoietic cells within the graft, including MSCs, may be largely responsible [17].

The potential role for MSCs in RDEB therapy was most recently supported by Alexeev and colleagues [26], who used a *Col7a1*^*–/–*^ mouse model to demonstrate that intradermal injections of wild-type MSCs could partially restore the basement membrane by increasing local C7 expression to 15% that of wild-type mice. However, we previously found that the use of wild-type MSCs as a stand-alone systemic infusion therapy was insufficient to rescue *Col7a1*^*–/–*^ mice from their typical early death, despite the ability of wild-type MSCs to express *Col7a1* mRNA, albeit at relatively low levels [27]. While these shortcomings may in part be due to the current inefficiencies of systemic MSC infusions, they are also confounded by the very short lifespan (2 to 3 days) of RDEB pups. Additionally, within murine RDEB models, simply increasing the number of transplanted MSCs to enhance cumulative C7 expression potentiates the odds of infusional toxicity, where entrapment of donor cells in pulmonary capillaries and/or peripheral organs results in recipient dysfunction. Thus, although intradermal injection of MSCs throughout affected body surfaces of RDEB patients would be an arduous process, the previous results from Alexeev and colleagues [26] provide evidence that if systemic infusions of MSCs are able to reach cutaneous areas in sufficient quantities, restoration of basement membrane integrity is a realistic outcome.

It may be possible to improve the efficacy and safety of allogeneic infusion protocols in the context of RDEB by utilizing a combined approach in which MSC migration to wounded tissue is enhanced and their transcription of *COL7A1* is upregulated, thereby increasing cumulative C7 secretion within recipient tissue. In theory, this could allow for a reduced immunomyeloablative conditioning regimen by taking advantage of the immunosuppressive properties of MSCs, while also promoting an increased functional utility for MSCs via increased *COL7A1* transcription.

With regards to an enhanced migratory capacity for MSCs, the CXCR4/stromal cell-derived factor 1-alpha (SDF-1α) axis, an interaction classically attributed to lymphocyte homing and development, has also been implicated in the recruitment of transplanted cells to injured tissue. Studies examining potential stem cell therapies for spinal cord injury [28] and myocardial infarction [29] have demonstrated the importance of the CXCR4/SDF-1α axis in this recruitment process. Furthermore, Jones and colleagues demonstrated that treating human fetal MSCs with SDF-1α *in vitro* resulted in a significant upregulation of *CXCR4* transcription, as well as an increase in the number of cell surface CXCR4^+^ cells [30]. This strategy led to improved transplantation outcomes in a model of osteogenesis imperfecta, and holds promise as a technique to improve the number of exogenous MSCs recruited to injured tissue in various disease models.

Furthermore, a prime example of how the immunosuppressive properties of MSCs can coincide with their ability to improve wound healing is demonstrated by tumor necrosis factor alpha (TNFα)-stimulated protein 6 (TSG-6). Expression of TSG-6 by MSCs has been associated with both improved wound healing and downregulation of macrophage proinflammatory signals at wounded tissue sites [31]. The role of TSG-6 in transplanted MSCs has also been highlighted by its anti-inflammatory properties and its ability to reduce infarct sizes in a model of myocardial infarction [32]. Transplantation therapies with the goal of healing wounded tissue and/or providing anti-inflammatory effects could thus potentially benefit from increasing the degree of TSG-6 expression within the population of transplanted cells.

Lastly, previous studies have demonstrated the feasibility of upregulating *COL7A1* transcription in fibroblasts [33, 34] and keratinocytes [35] by incubating cells in the presence of cytokines such as TNFα and transforming growth factor beta (TGFβ). The upregulatory function that TGFβ has on COL7A1 expression has been characterized previously [36, 37]. Additionally, Knaup and colleagues found that expression of *COL7A1* in the RDEB cell lines was increased and attributable to elevated TGFβ levels in the local environment [38]. However, it remains to be seen whether these findings can be applied to MSCs; that is, whether MSCs can be induced to upregulate *COL7A1* expression, and furthermore whether increased *COL7A1* expression can be coupled with enhanced MSC migration and immunosuppression as a model for improved transplantation efficacy in RDEB. In the present study, we demonstrate that an *in vitro* cytokine preconditioning protocol can simultaneously upregulate *Cxcr4*, *Tsg-6* and *Col7a1* expression within murine MSCs. We also evaluate whether this approach can serve as a realistic addition to current stem cell infusion protocols aimed at treating RDEB patients.

## Materials and methods

### Isolation and culture of murine mesenchymal stem cells

MSCs were extracted from compact bone of healthy mice between the ages of 2 and 3 weeks using the protocol and characterizations described by Zhu and colleagues [39]. Cells were cultured in alpha minimum essential medium (αMEM) +10% fetal bovine serum +100 U/ml penicillin/streptomycin. Culture medium was changed every 2 or 3 days, and plastic-adherent cells were passaged at 70 to 80% confluence using 0.25% trypsin–ethylenediamine tetraacetic acid. Cells from passages 2 to 4 were used in all experiments. All animal studies were approved by the University of Minnesota Institutional Animal Care and Use Committee.

### Reagents

Ligands, cytokines, and antagonists used within the preconditioning protocol and related experiments were purchased from R&D Systems (Minneapolis, MN, USA): recombinant mouse CXCL12/SDF-1α, recombinant mouse TGFβ2, recombinant mouse TNFα, and AMD3100.

### RNA extraction, reverse transcription, and quantitative polymerase chain reaction

RNA was extracted using an RNeasy Mini Kit and RNase-Free DNase Set (Qiagen, Venlo, the Netherlands) according to the manufacturer’s protocol. RNA concentrations were quantified using a NanoDrop system (Thermo Fisher Scientific, Waltham, MA, USA). All samples used in downstream experiments had an absorbance_260/280_ ratio exceeding 2.00, and ribonucleic acid concentrations were diluted to 50 to 100 ng/μl prior to cDNA synthesis. cDNA was synthesized using a High Capacity cDNA Reverse Transcription Kit (Applied Biosystems, Foster City, CA, USA). Quantitative polymerase chain reaction (qPCR) was carried out using a StepOnePlus Real-Time PCR system (Applied Biosystems). SYBR Green Master Mix reagent (Life Technologies, Grand Island, NY, USA) was used for a fluorescent probe according to the manufacturer’s guidelines. Primers for *Col7a1* consisted of 5′-TGGTAACAACCTCGGCACAG-3′ (forward) and 5′-AAGTCTGGGCCTCACGAATG-3′ (reverse). Primers for *Tsg-6* consisted of 5′-GCTCACGGATGGGGATTCAA-3′ (forward) and 5′-TTGTAGGTTGCGAGACGACC-3′ (reverse). Primers for *Cxcr4* consisted of 5′-CGGCTGTAGAGCGAGTGTTG-3′ (forward) and 5′-CATCAACTGCCCAGAAGGGG-3′ (reverse). Primers for *GAPDH* consisted of 5′-CCAGCAAGGACACTGAGCAA-3′ (forward) and 5′-CCCTAGGCCCCTCCTGTTAT-3′ (reverse). All qPCR reactions were carried out in triplicate in a total reaction volume of 20 μl (8 μl RNase-free water, 10 μl of 2× SYBR Green Master Mix, 0.5 μl each forward and reverse primers, and 1 μl cDNA). Reaction times and temperatures for all qPCR reactions were as follows: initial 10-minute hold for enzyme activation (95°C) followed by 40 cycles of 15-second denaturing (95°C), 30-second annealing (53°C), and 30-second extension (60°C). qPCR data were analyzed using ExpressionSuite Software (Applied Biosystems) according to the comparative cycle threshold (2^–∆∆CT^) method. PCR for purposes of gel electrophoresis was carried out using AmpliTaq DNA polymerase reagents (Applied Biosystems) according to the manufacturer’s instructions.

### Enzyme-linked immunosorbent assays

Sandwich enzyme-linked immunosorbent assays (ELISAs) for detection of secreted C7 were performed using an anti-mouse C7 ELISA kit (CUSABIO, Wuhan, China) according to the manufacturer’s protocol. For each experiment, 2 × 10^5^ cells were split evenly into two flasks and incubated in αMEM. Treated cells were exposed to 15 ng/ml TGFβ +30 ng/ml TNFα. At 48 hours, medium was removed and frozen at –80°C until ELISAs were carried out.

### *In vitro*chemotaxis assay

*In vitro* chemotaxis assays were performed using a 12-well chemotaxis chamber (Neuro Probe Inc., Gaithersburg, MD, USA). GFP-expressing cells were lifted using 0.25% trypsin–ethylenediamine tetraacetic acid and allowed to settle in a 1 ml suspension of αMEM for 1 hour prior to chemotaxis experiments. Cells were suspended at a concentration of 5 × 10^5^/ml, such that approximately 50,000 cells were placed into the 100 μl top compartments. Bottom wells were filled with αMEM + varying concentrations of SDF-1α. For blocking controls, cells were incubated for 1 hour in 100 μg/ml AMD3100, a potent CXCR4 receptor antagonist. Following the assays, the nonmigrated surfaces of the 25 × 80 mm polycarbonate filters were washed in phosphate-buffered saline, and the migrated surfaces were fixed in 10% neutral buffered formalin. Cells were visualized under a fluorescent microscope using a FITC filter and counted three times per well at 200× (total magnification).

### Flow cytometry

Flow cytometry experiments were carried out on a FACSCanto system (BD Biosciences, San Jose, CA, USA) and analyzed using FlowJo (Tree Star Inc., Ashland, OR, USA) and FCX Express 4 Research Edition (De Novo Software, Los Angeles, CA, USA). Prior to extracellular staining, cells were lifted using 0.25% trypsin–ethylenediamine tetraacetic acid and allowed to settle in a 1 ml suspension of αMEM for 1 hour. Extracellular CXCR4 was detected using 1 μg/100 μl APC-tagged rat monoclonal anti-mouse CXCR4 antibody with 1 μg/100 μl APC-tagged rat IgG2b-′ antibody used as an isotype control (BD Biosciences). All extracellular staining included an initial Fc block using a purified rat monoclonal anti-mouse CD16/CD32 antibody at 0.5 μg/100 μl (eBioscience, San Diego, CA, USA).

### Data analysis

Differences between measured variables were conducted using a two-tailed Student’s *t* test, with *P* <0.05 considered significant.

## Results

### Effect of preconditioning duration on *Col7a1*and *Tsg-6*mRNA expression

**Figure 1:**
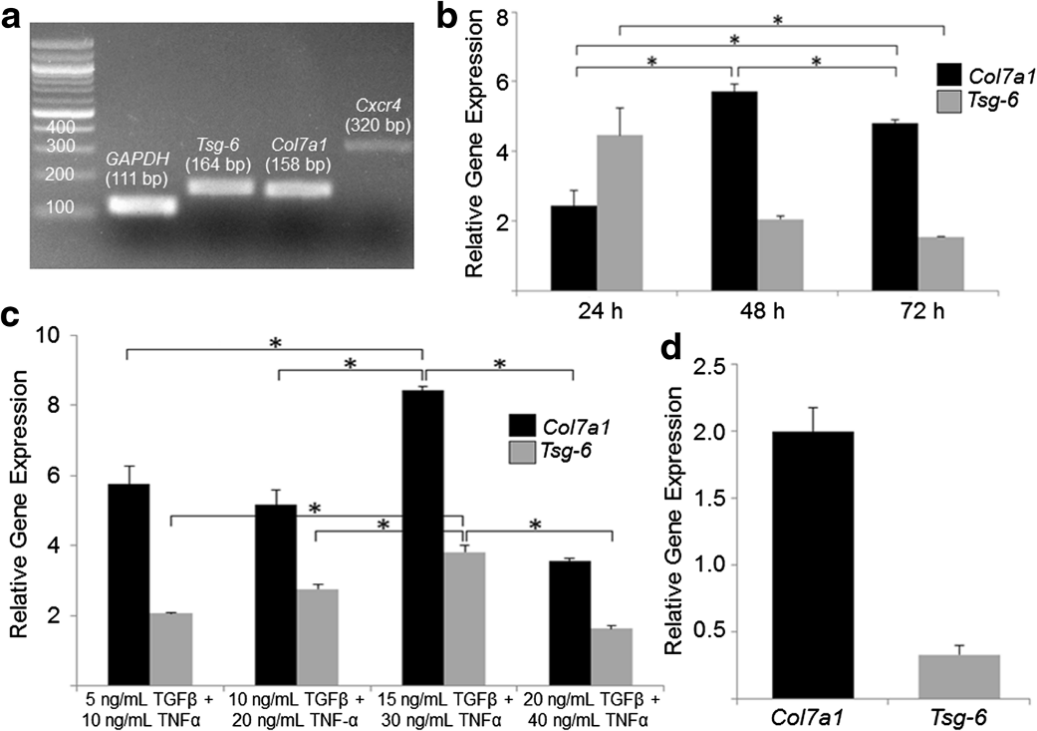
**Cytokine preconditioning induces simultaneous upregulation of** ***Col7a1*** **and** ***Tsg-6*** **mRNA expression in mesenchymal stem cells. (a)** Untreated mesenchymal stem cells (MSCs) exhibit detectable baseline expression of *Col7a1*, *Tsg-6*, and *Cxcr4*. **(b)** MSCs were treated with 10 ng/ml transforming growth factor beta (TGFβ) +20 ng/ml tumor necrosis factor alpha (TNFα) for 24, 48, or 72 hours. Quantitative polymerase chain reaction (qPCR) was performed for *Col7a1* and *Tsg-6* expression in treated groups relative to untreated MSCs. **(c)** MSCs were treated across concentration gradients of TGFβ and TNFα for 48 hours. qPCR was performed for *Col7a1* and *Tsg-6* expression in treated groups relative to untreated MSCs. **(d)** MSCs were treated with 15 ng/ml TGFβ +30 ng/ml TNFα for 48 hours. Cells were transferred to an alpha minimum essential medium-only environment for a subsequent 48 hours, after which qPCR was performed for *Col7a1* and *Tsg-6* expression in treated groups relative to untreated MSCs. All qPCR values in (b) to (d) were normalized against endogenous glyceraldehyde 3-phosphate dehydrogenase (*GAPDH*) expression. All qPCR experiments were run in triplicate and across two experimental groups per condition. Data presented as mean ± standard deviation. *P <0.05 by Student’s *t* test.

To investigate whether murine MSCs are capable of upregulating transcription of *Col7a1* and *Tsg-6*, cells were treated with 10 ng/ml TGFβ +20 ng/ml TNFα in αMEM, incubated for 24, 48, or 72 hours, and compared with untreated controls. Following the designated incubation periods, RNA was extracted, reverse transcribed, and subjected to qPCR. The observed relative quantification values across two experiments are displayed in Figure 1b. As shown, increased transcription of both *Col7a1* and *Tsg-6* was observed across all three time points, demonstrating that MSCs can upregulate transcription of these two genes via exposure to cytokine preconditioning. With regards to a time-dependent effect of preconditioning, *Col7a1* transcription was significantly higher at 48 hours (5.7-fold increase ±0.20) relative to 24 and 72 hours, while *Col7a1* transcription at 72 hours was also significantly higher than at 24 hours. *Tsg-6* transcription was highest at 24 hours (4.5-fold increase ±0.79) and significantly higher than at 72 hours but not at 48 hours.

### Effect of preconditioning dosage on *Col7a1*and *Tsg-6*mRNA expression

To determine whether a dose–response effect exists with regards to strength of cytokine exposure and subsequent changes in *Col7a1* and *Tsg-6* transcription, cells were treated for 48 hours in the presence of varying cytokine concentrations and compared with untreated controls (Figure 1c). Cells treated with 15 ng/ml TGFβ +30 ng/ml TNFα showed the greatest increase in both *Col7a1* (8.4-fold increase ±0.12) and *Tsg-6* (3.8-fold increase ±0.19) transcription, and these changes were significantly higher than in the other three treatment groups. There was no significant difference in *Col7a1* transcription between the 5 ng/ml TGFβ +10 ng/ml TNFα and the 10 ng/ml TGFβ +20 ng/ml TNFα groups, while a significant increase and decrease was seen below and above the 15 ng/ml TGFβ +30 ng/ml TNFα group, respectively. These results, taken together with those shown in Figure 1b, demonstrate that treating cells with 15 ng/ml TGFβ +30 ng/ml TNFα for 48 hours elicits the greatest fold increase in *Col7a1* transcription, and this protocol was used in all subsequent experiments.

### Preconditioning effects after removal of cytokines

To determine how persistent the preconditioning effects are with regards to *Col7a1* and *Tsg-6* mRNA expression, cells were treated with 15 ng/ml TGFβ +30 ng/ml TNFα for 48 hours, washed with phosphate-buffered saline, and placed in αMEM for 48 hours as a cytokine-free environment. Figure 1d shows that after being removed from the preconditioning environment for 48 hours, there was still a twofold increase (±0.17) in *Col7a1* mRNA levels relative to untreated cells. Interestingly, *Tsg-6* mRNA expression appeared to be downregulated once removed from the preconditioning environment. These results suggest that while *Col7a1* upregulation can be maintained for at least 48 hours following removal of cytokine stimuli, the effects on *Tsg-6* upregulation are more transient and revert to a downregulated state within 48 hours of cytokine removal. It should be noted that, as seen in Figure 1b, both *Col7a1 and Tsg-6* transcription could be held in the upregulated state for at least 72 hours as long as the preconditioning environment was present, but, as the results in Figure 1d demonstrate, once cells were removed from the preconditioning environment the upregulatory effects on *Tsg-6* transcription appear to be reversed in the absence of extracellular cytokines.

### Effects of preconditioning on type VII collagen protein secretion

**Figure 2:**
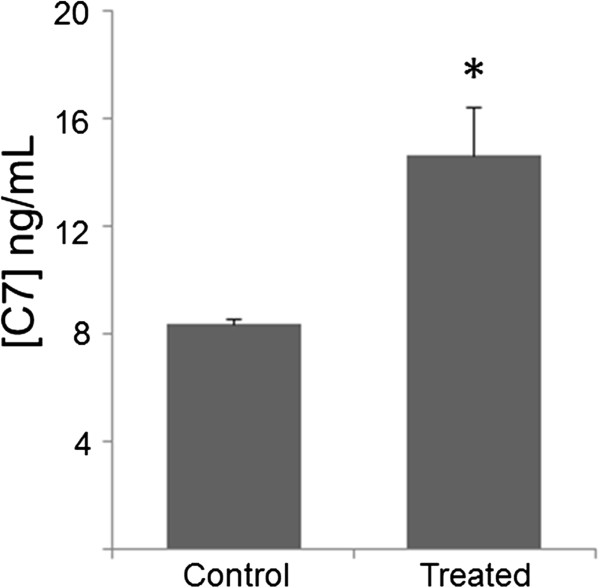
**Cytokine preconditioning results in increased type VII collagen protein secretion.** Mesenchymal stem cells (MSCs) were treated with 15 ng/ml transforming growth factor beta +30 ng/ml tumor necrosis factor alpha for 48 hours. Following this incubation period, culture medium was extracted and subjected to sandwich enzyme-linked immunosorbent assay (ELISA) analysis. Culture medium from treated groups was compared with that of untreated MSCs. ELISA experiments were carried out across two biological groups per condition (10^5^ cells per group). Data presented as mean ± standard deviation. *P <0.005 by Student’s *t* test. C7, type VII collagen protein.

To demonstrate whether the transcriptional upregulation of *Col7a1* seen following cytokine preconditioning corresponds to increased secretion of C7, a sandwich ELISA was performed to compare the culture medium of untreated cells with cells treated for 48 hours with 15 ng/ml TGFβ +30 ng/ml TNFα. Prior to the 48-hour incubation period, 2 × 10^5^ cells were split evenly into two flasks for each of the two experimental and control groups. As shown in Figure 2, treated cells showed a significantly higher level of C7 secretion relative to untreated cells and an approximate 70% increase above baseline (14.4 ± 1.6 vs. 8.3 ± 0.17 ng/ml; *P* <0.005).

### Simultaneous upregulation of *Cxcr4*, *Col7a1*, and *Tsg-6*

**Figure 3:**
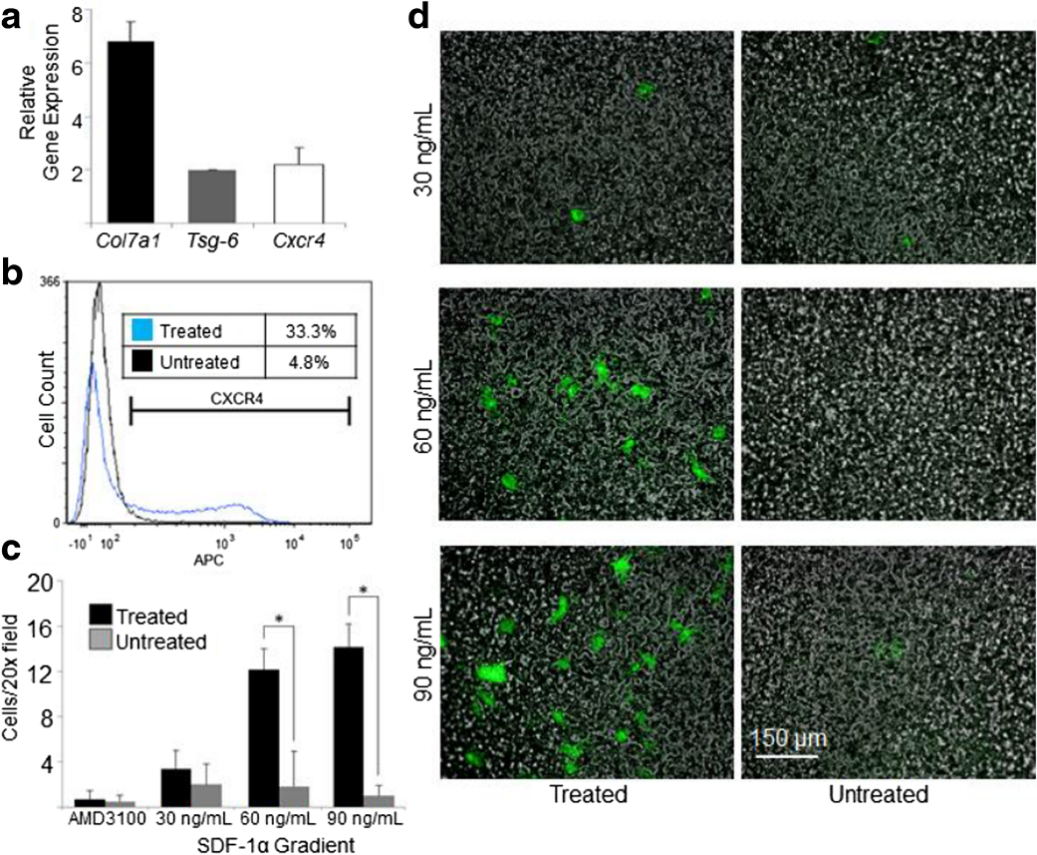
**Addition of SDF-1α to the preconditioning protocol induces simultaneous upregulation of** ***Col7a1, Tsg-6,*** **and** ***Cxcr4*** **mRNA expression.** Mesenchymal stem cells (MSCs) were treated with 15 ng/ml transforming growth factor beta +30 ng/ml tumor necrosis factor alpha for 48 hours. At 47 hours, cells were exposed to 30 ng/ml stromal cell-derived factor 1-alpha (SDF-1α) for 1 hour. **(a)** Quantitative polymerase chain reaction (qPCR) was performed for *Col7a1*, *Tsg-6*, and *Cxcr4* expression in treated cells relative to untreated MSCs. qPCR values were normalized against endogenous glyceraldehyde 3-phosphate dehydrogenase expression, and experiments were run in triplicate and across two experimental groups. **(b)** Flow cytometry was performed to assess cell surface CXCR4 expression in treated versus untreated cells. **(c)** Chemotaxis assay results of treated versus untreated cells: 50,000 GFP-expressing cells were placed in each top well, while increasing SDF-1α gradients were used in the bottom wells. For blocking controls, treated and untreated cells were incubated in presence of 100 μg/ml AMD3100 for 1 hour and exposed to a 90 ng/ml SDF-1α concentration gradient during the assay. Experiments were run in duplicate. **(d)** Representative fluorescent microscopy images of the chemotaxis membrane following the assay. Data presented as mean ± standard deviation. *P <0.05 by Student’s *t* test.

The importance of the CXCR4/SDF-1α chemokine axis within the context of stem cell transplantation and migration to injured tissue has been demonstrated across several disease models [28–30]. Having shown simultaneous upregulation of *Col7a1* and *Tsg-6* mRNA expression, we next aimed to incorporate *Cxcr4* upregulation as part of the preconditioning protocol. To achieve this, cells were treated with 15 ng/ml TGFβ +30 ng/ml TNFα for 48 hours as described above, after which 30 ng/ml SDF-1α was introduced for 1 hour. Figure 3a demonstrates that, under this protocol, upregulation of all three genes could be achieved simultaneously, and that *Cxcr4* mRNA levels were 2.2-fold higher than in untreated cells. While *Col7a1* expression here was not significantly different from that in cells treated with 15 ng/ml TGFβ +30 ng/ml TNFα for 48 hours without the 1-hour SDF-1α treatment (Figure 1c), *Tsg-6* expression was significantly less (3.8-fold vs. 2.0-fold, *P* <0.05) in the presence of SDF-1α treatment, although still twofold higher than in untreated cells and comparable with levels seen in the other treatment gradients shown in Figure 1c.

To assess the physiologically relevant utility of this protocol – that is, whether preconditioning could also upregulate cell surface CXCR4 protein – treated cells were examined using flow cytometry (Figure 3b) and were found to exhibit a 28.5% increase in the cell surface CXCR4 signal relative to untreated cells. Next, a chemotaxis chamber was used to determine whether this increase in cell surface CXCR4 would result in improved migratory capabilities toward an SDF-1α gradient. As displayed in Figure 3c, treated cells showed a significantly greater migratory response toward SDF-1α gradients of 60 and 90 ng/ml, but not 30 ng/ml. Importantly, it is worth noting that, similar to the report by Potapova and colleagues [40], our attempts at characterizing cell surface CXCR4 expression of cells brought directly from monolayer conditions to flow cytometry experiments generally yielded an undetectable CXCR4 signal (data not shown), while cells that were subjected to a 1-hour resettling period in a cell suspension environment during SDF-1α treatment yielded the results described herein.

## Discussion

The results presented here demonstrate for the first time, to the best of our knowledge, upregulation of *Col7a1* mRNA and C7 expression in MSCs using an exogenous preconditioning protocol. Additionally, we present the feasibility of a three-tiered preconditioning model to improve the efficacy of transplanted MSCs in the context of RDEB therapy. This model incorporates: an improved chemotactic response by MSCs toward an SDF-1α gradient (via *Cxcr4* upregulation and increased cell surface CXCR4 expression) as a surrogate for homing to injured tissue; an increased functional role for MSCs once present in tissue (via increased *Col7a1* and C7 expression); and a more potent immunosuppressive arsenal and wound-healing response of MSCs via upregulation of *Tsg-6* expression. Given the demonstrated ability of implementing only three cytokines (TGFβ, TNFα, and SDF-1α) to induce a simultaneous upregulation in *Col7a1*, *Tsg-6*, and *Cxcr4*, we believe that this protocol represents a very straightforward yet potentially high-yield approach for improving the efficacy of MSCs in the context of RDEB transplantation, either as a supportive role within HCT or as a potential standalone therapy. We also demonstrate the feasibility and broad applicability of preconditioning protocols, whereby transplanted cells are rendered more functionally suitable *in vitro* for the specific disease of interest prior to transplantation.

To appreciate the physiologic significance of a sixfold to eightfold increase in *Col7a1* expression by MSCs, it is necessary to compare expression levels across various cell types. We previously provided a characterization of baseline *Col7a1* expression across murine bone marrow and stem cell lineages, and found MSCs to exhibit an approximate 15-fold greater expression profile than whole bone marrow cells as well as CD150^+^/48^–^ and Lin^–^ subsets of the bone marrow population [27]. Based on these previous characterizations, a sixfold to eightfold increase in baseline *Col7a1* expression of MSCs, as demonstrated throughout the present study, would place them at about one-third the expression level of multipotent adult progenitor cells, and at 18% of the relative *Col7a1* expression found in wild-type skin. At the protein level, Alexeev and colleagues found that intradermal injections of wild-type MSCs into a mouse model of RDEB resulted in C7 expression at 15% that of wild-type levels [26]. Incorporating the approximate 70% increase in C7 secretion we observed under our current preconditioning regimen, this would bring C7 levels toward the 30% of the amount of wild-type C7 that is believed to be adequate for preventing blistering in the context of RDEB [41]. Thus, it is reasonable to suggest that preconditioned MSCs would be capable of supplying the necessary C7 to facilitate significant restoration of the DEJ following transplantation.

To address the transiency of preconditioning effects observed in the present study, we demonstrate that *Col7a1* expression can be held in the upregulated state for at least 72 hours in the presence of cytokines. We also demonstrate that an upregulated state of *Col7a1* expression can be held for at least 48 hours following removal of cytokine stimuli, albeit at lower levels than seen in the presence of preconditioning. A legitimate question thus arises regarding whether the increase in *Col7a1* would be too transient to establish any significant change following transplantation. Here we wish to emphasize the Trojan horse aspect of preconditioning with regards to delivery of C7 to cutaneous sites, where the increase in *Col7a1* expression seen pre-transplant would provide an initial restorative benefit, after which MSCs would be expected to resume baseline C7 secretion, which Alexeev and colleagues have shown to be approximately 15% that of wild-type DEJ [26]. Based on our current results, the transition from an upregulated state to a baseline state would be expected to occur after at least 48 hours, and perhaps even longer depending on the cytokine milieu present in RDEB skin [42]. Importantly, RDEB cells have been shown to exhibit increased expression of TGFβ and *COL7A1*[38], albeit dysfunctional *COL7A1*, and thus it is logical that preconditioned wild-type MSCs would continue to display elevated C7 secretion once in the RDEB environment via elevated TGFβ *in vivo*. Cytokine preconditioning of MSCs may thus provide an added initial influx of C7 to cutaneous sites, followed by a probably lessened but continual secretion of C7. Future *in vivo* studies will be necessary to examine whether systemic infusions of preconditioned MSCs are capable of bringing C7 levels at the DEJ in RDEB skin to levels sufficient for long-term cutaneous repair.

It is worth highlighting the downregulation in *Tsg-6* expression (3.8-fold vs. 2.0-fold) we observed when going from the 48-hour treatment of 15 ng/ml TGFβ +30 ng/ml TNFα (Figure 1c) to the addition of 30 ng/ml SDF-1α for 1 hour (Figure 3a). It is conceivable that SDF-1α, a chemokine implicated in the migration of proinflammatory cells, may exert feedback inhibition on anti-inflammatory signals such as TSG-6. Although both *Cxcr4* and *Tsg-6* genes could simultaneously be brought to the upregulated state, it appears that in the context of a pre-transplantation protocol, the addition of SDF-1α to the preconditioning regimen would be at the expense of partial loss of *Tsg-6* expression. Given the improved migrational performance seen with the addition of SDF-1α, however, increased cell surface CXCR4 protein at the expense of partially dampened *Tsg-6* expression may be worthwhile during the transplantation window, as wounded cutaneous sites (for example, RDEB skin) experienced by MSCs following transplantation would provide an additional stimulus for prolonged *Tsg-6* upregulation *in vivo*[31].

Furthermore, it is worth noting that although we were able to observe a 2.2-fold increase in *Cxcr4* expression using the protocol described by Jones and colleagues [30], we were unable to attain the degree of upregulation described in their original findings (approximately fivefold). A probable explanation for this difference is the source of cells used, as human fetal MSCs were used by Jones and colleagues while MSCs from mice aged 2 to 3 weeks were used in the present study. Potapova and colleagues also described an internalization of CXCR4 protein in response to SDF-1α exposure in human MSCs [40], and here we report an increase in cell surface CXCR4. However, the difference in responses is probably due to the vastly greater SDF-1α concentrations used by Potapova and colleagues (1 μg/ml vs. 30 ng/ml in the present study) and the differing effects of such concentrations on sensitization of cell surface CXCR4 and shunting of the chemokine receptor toward intracellular pools.

Recently, the work by Lin and colleagues has provided an exciting demonstration of the clinical possibilities afforded by manipulating the CXCR4/SDF-1α axis [43]. The administration of AMD3100 (a CXCR4 antagonist) and low-dose tacrolimus resulted in liberation of bone marrow cells into the circulation and improved wound healing at cutaneous sites. While these results could have implications across many clinical contexts, they also highlight the importance of SDF-1α expression at wounded tissue sites and its role in recruiting CXCR4-expressing bone marrow-derived cells, as blocking SDF-1α using intradermal antibody injections resulted in a loss of wound healing benefits. Looking beyond the CXCR4/SDF-1α axis, future attempts at exogenously upregulating the CCR10 expression of MSCs prior to transplantation may also prove to be a valuable approach, as the CCR10/CCL27 axis has been implicated in improved targeting of MSCs to cutaneous sites [42].

### Transforming growth factor beta: more than just fibrosis

Like many cytokines, TGFβ has numerous attributed roles in a variety of contexts. One of its most widely known functions is as an anti-inflammatory and profibrotic stimulus. Specifically, TGFβ released from macrophages during an inflammatory response is known to promote myofibroblast differentiation as part of the wound repair and profibrotic process [31]. Additionally, culturing MSCs in the presence of TGFβ has previously been shown to upregulate levels of α-smooth muscle actin [44], a marker attributed to but not specific for myofibroblasts. If these two properties are loosely connected, an association between TGFβ-treated MSCs adopting a myofibroblast-like phenotype and a subsequent progression to a profibrotic state may be drawn. However, increased α-smooth muscle actin and associated rates of contractile activity are not limited to fibrotic processes, and in fact are thought to be an important mechanism in tissues that are actively employing new extracellular matrix and/or attempting to increase tissue strain [45]. While this has been demonstrated in settings such as ligament repair [46], it may also be involved at the DEJ in RDEB skin following incorporation of new C7 into the tissue architecture. Additionally, preconditioning of MSCs using a cytokine cocktail that included TGFβ was shown to be beneficial in restoring cardiac function in a murine model of myocardial infarction [47]. Previous reports of TGFβ-mediated increases of α-smooth muscle actin and contractility in MSCs should thus not be used synonymously with a profibrotic process, but instead should be looked at as a mechanism that can occur in a variety of physiologic contexts. Of course, future *in vivo* studies are needed to assess whether the contextual benefits of TGFβ preconditioning outweigh any profibrotic changes that may accumulate prior to the MSCs reverting back to the baseline state, where they have previously been shown to have a beneficial effect on the cutaneous environment in the RDEB phenotype [26].

Prolonged TGFβ signaling has also been implicated in the context-dependent procarcinogenic transformation of MSCs in certain cancer pathologies. For example, MSCs cultured for 21 days in the presence of TGFβ as part of tumor-conditioned medium were shown to increase expression of procarcinogenic factors [48]. Culturing MSCs with tumor-conditioned medium for 16 days was also shown to promote transition of the cells into tumor-associated fibroblasts, which are associated with various protumorigenic and epithelial-to-mesenchymal functions [49]. Conversely, inhibition of TGFβ signaling in MSCs exposed to tumor-secreted factors led to increased proinflammatory responses of MSCs to the tumor microenvironment [50]. Thus, while TGFβ is certainly an important cytokine for interactions between dysplasias and local MSCs, its role is probably context dependent and cell line dependent, and is thought to serve both tumor suppressive and pro-epithelial-to-mesenchymal functions in different settings [51].

Patients with RDEB experience drastically increased rates of squamous cell carcinoma (SCC), and this process may involve the known increased TGFβ signaling found in RDEB SCC skin [38]. However, since TGFβ is also elevated in non-SCC RDEB skin, Knaup and colleagues reflect that the increased TGFβ in this setting may also be an attempt to call for increased *COL7A1* expression rather than as part of a strict carcinogenic process. They also point out that increased TGFβ by itself is unlikely to cause malignant consequences, while concomitant mutations and stage of tumorigenesis during exposure to elevated TGFβ signaling seems to be more significant [38, 52]. Of course, cellular therapies for RDEB involving any aspect of TGFβ and other cytokine signaling should seriously consider whether an added risk for SCC may develop. Given that the involved pathways probably require prolonged time intervals and multifaceted signals to develop, the 48-hour preconditioning protocol presently used that involves isolated TGFβ and TNFα signals would not be expected to increase risk for SCC following transplantation. Additionally, the chronic inflammatory state and dysfunctional cutaneous environment associated with RDEB are thought to largely drive the increased risk for SCC [38], and the presented therapy would seek to limit chronic inflammation via stabilization of the DEJ and thus reduce overall cancer progression. However, it is unclear what the negative consequences may be following the introduction of cytokine-treated MSCs into RDEB patients, specifically those in which epithelial dysplasia has previously been established. Future studies will be necessary to elucidate whether this therapeutic approach may elevate risk for SCC in recipients with or without prior epithelial dysplasia.

### Emerging potential of preconditioning

The concept of preconditioning MSCs prior to transplant has up to this point been largely focused on the settings of myocardial infarction (see review by Li and colleagues [53]) and ischemic stroke (see review by Yu and colleagues [54]). Although the degree of transplanted cell death in these ischemic environments has represented a barrier to their therapeutic potential, *in vitro* hypoxic preconditioning has been used as a method to enhance MSC graft survival post transplant [55]. In addition to promoting cell survival, preconditioning in ischemic disease has also been shown to prove functionally useful. For instance, *in vitro* treatment of MSCs using oxidative stress signals led to upregulation of various cardiogenic factors [56], and this method may show promise for future myocardial infarction infusion protocols. Herrmann and colleagues found that preconditioning with TGFα led to an enhanced cardioprotective role for MSCs [57], and MSCs preconditioned with a cytokine cocktail, including TGFβ, were shown to be beneficial for restoring cardiac function in a model of myocardial infarction [47]. Furthermore, enabling MSCs to be better migrators toward injured tissue is another application of preconditioning, as shown previously by Jones and colleagues via upregulation of CXCR4 expression in a model of osteogenesis imperfecta [30]. Several aspects of MSC functionality – whether graft survival, migration, or disease modification – have thus been demonstrated to improve under preconditioning protocols. Considering that our overall knowledge of MSCs is still in its relative infancy, even more so is our understanding of their potential applications toward therapy. As researchers continue to target MSCs as candidates for cell-based therapies in the future, the concept of preconditioning is something that should be considered for investigation. Since some form of *in vitro* expansion is required as an intermediate step between harvesting and transplant due to the relative low frequency of MSCs at extraction sites, the addition of preconditioning protocols does not require extensive time or effort, and the advantages gained from this application could have extraordinary potential.

### Clinical strategies for approaching recessive dystrophic epidermolysis bullosa therapy

There are several promising approaches on the horizon for attaining improved outcomes in RDEB patients. First, there exist considerable efforts to further modify stem cell transplantation techniques that have previously been shown to ameliorate the RDEB phenotype [15]. While the exact mechanism as to how HCT is capable of producing these results has yet to be fully elucidated, it is thought that nonhematopoietic cells within the graft, including MSCs, may be largely responsible [17]. This hypothesis is supported in part by findings that bone marrow-derived MSCs can give rise to epithelial progenitors that promote regeneration and restoration of C7 within grafted C7-null skin [58], and also by evidence that MSCs are directly capable of restoring partial DEJ function in RDEB skin [26]. The ability to exogenously upregulate *COL7A1* and C7 expression in MSCs in the pre-transplant period, as demonstrated here, thus supports a larger and more defined role for MSCs within the overall transplantation approach toward RDEB therapy in the future. The use of stem cell transplantation is not without its hazards, however, as the intensive immunosuppressive regimen required for such a procedure is an additional stressor to RDEB patients. An additional benefit of expanding the role for MSCs in this context may thus allow for a less intensive immunomyeloablative protocol in the pre-transplant and post-transplant periods by taking advantage of the inherent immunosuppressive properties of MSCs. With regards to how this preconditioning method may impact screening and harvesting protocols for allogeneic transplants, existing methods such as haplotyping, extraction, expansion, and fluorescence-activated cell sorting would largely go unchanged (see review by Ikebe and Suzuki [59] for overview of MSC collection and expansion protocols). Of course, the incorporation of a preconditioning regimen would require an additional step within the expansion phase of cell preparation, but would otherwise not be expected to complicate existing protocols. Whether additional safety concerns would be introduced during the infusion window by way of applying exogenous cytokines during cell culture expansions is something that will need to be addressed in future *in vivo* animal and human studies.

Second, the use of intradermal fibroblast injections as a method for treating RDEB has transitioned into the setting of human studies [10, 60]. These methods have been shown to improve wound healing in ulcerated areas of patients’ skin and to promote increased presence of C7 at the DEJ. The current understanding of how injected fibroblasts exert these beneficial effects is via upregulating endogenous production of mutant C7 [61]. Thus, while these techniques may prove useful in RDEB patients with some degree of functional baseline C7 production, they may not attain benefits in patients with complete absence of *COL7A1* expression. Taking into consideration that fibroblasts have previously been shown to upregulate *COL7A1* expression via *in vitro* cytokine treatments [33, 34], the idea of preconditioning cells prior to transplantation, as demonstrated in the present study with regards to MSCs, is something that warrants investigation in other transplantation modalities such as intradermal fibroblast injections. As with other therapeutic strategies in the context of RDEB, however, intradermal fibroblast injections are not without their limitations. The need for multiple injections across different areas of skin and the questions surrounding the half-life of efficacy for each injection are variables that will need to be addressed in the future, and that also highlight the benefits of transitioning to systemic allogeneic fibroblast infusions as a potential related therapeutic modality.

Third, there has been considerable attention placed on the idea of using C7 as a therapeutic strategy for treating RDEB patients. This approach began with the use of intradermal recombinant C7 injections [12, 13], which were shown to reverse the RDEB phenotype in grafted skin as well as in an RDEB mouse model. Given that the use of intradermal injections could be limited by the diffusing capacity of C7 and the large surface area of RDEB lesions, as well as the inability to reach mucosal lesions (for example, of the esophagus), the use of systemic intravenous infusions of soluble C7 have now come into focus [14]. Initial reports of this approach demonstrated an incorporation of injected C7 into the DEJ of RDEB skin grafts and improved dermal–epidermal integrity. While it is likely that this approach will one day translate into improved outcomes in human RDEB patients, the use of systemic C7 injections, much like stem cell transplantation and intradermal fibroblast injections, is also not without its limitations. For instance, although C7 exhibits a relatively long half-life of several months [62], in the absence of an endogenous producer of functional C7 it is conceivable that an individual with RDEB would require lifelong rounds of injection for a sustainable therapy to manifest itself. Additionally, it will be important to determine whether certain recipients may be at risk for developing immunity against injected C7. Recently, it has been demonstrated that anti-C7 antibodies may be relatively common among RDEB patients and that most may be nonpathogenic [63]. However, in the event that an antibody response does occur following injections, this approach may also warrant some degree of immunosuppressive modulation.

Each of the strategies for approaching RDEB therapy described above have several advantages and numerous obstacles. The effect of preconditioning on cells prior to transplant, specifically in terms of *COL7A1* upregulation as described here, would not only be beneficial in the context of bone marrow and cord blood transplantations for RDEB therapy, but could also prove valuable with regards to stromal cell (mesenchymal and fibroblast) therapies.

## Conclusions

To our knowledge, we demonstrate for the first time an upregulation of *Col7a1* mRNA and C7 expression in MSCs using an exogenous preconditioning protocol. By using a regimen of TGFβ, TNFα, and SDF-1α, MSCs are capable of simultaneously upregulating *Col7a1*, *Tsg-6*, and *Cxcr4* expression. This three-tiered approach renders MSCs more functionally equipped for treating RDEB via increased C7 secretion, more potent immunosuppressants and wound-healers via upregulated *Tsg-6*, and better migrators toward injured tissue via enhanced cell surface CXCR4 expression. HCT has previously been shown to ameliorate the RDEB phenotype in pediatric patients, and this response is thought to be partially attributable to MSCs within the graft [17]. Additionally, MSCs have been shown to restore C7 at the DEJ in a mouse model of RDEB [26]. These previous findings, along with our current presented data, suggest that preconditioned MSCs represent a feasible methodology for approaching systemic RDEB therapy. Ongoing and future *in vivo* studies and clinical trials involving allogeneic transplants for RDEB may benefit from analyzing the utility of such preconditioning protocols, and whether they provide an improvement over the effects seen with unconditioned cells.

## Abbreviations

αMEM: alpha minimum essential medium
C7: type VII collagen protein
DEJ: dermal–epidermal junction
ELISA: enzyme-linked immunosorbent assay
HCT: hematopoietic cell transplantation
MSC: mesenchymal stem cell
qPCR: quantitative polymerase chain reaction
RDEB: recessive dystrophic epidermolysis bullosa
SCC: squamous cell carcinoma
SDF-1α: stromal cell-derived factor 1-alpha
TGFβ: transforming growth factor beta
TNFα: tumor necrosis factor alpha
TSG-6: tumor necrosis factor alpha-stimulated protein 6.
